# Retraction Note: Mutant Runx2 regulates amelogenesis and osteogenesis through a miR-185-5p-Dlx2 axis

**DOI:** 10.1038/s41419-025-07762-2

**Published:** 2025-06-17

**Authors:** Huaiguang Chang, Yue Wang, Haochen Liu, Xu Nan, Singwai Wong, Saihui Peng, Yajuan Gu, Hongshan Zhao, Hailan Feng

**Affiliations:** 1 Department of Prosthodontics, Peking University School and Hospital of Stomatology & National Engineering Laboratory for Digital and Material Technology of Stomatology & Beijing Key Laboratory of Digital Stomatology, Beijing, China; 2 Department of Medical Genetics, Peking University Health Science Center, Beijing, China; 3 Peking University Center for Human Disease Genomics, Peking University Health Science Center, Beijing, China

Retraction Note to: *Cell Death & Disease* 10.1038/s41419-017-0078-4, published online 14 December 2017

The Editors-in-Chief have retracted this article. After publication, concerns were raised regarding highly similar images in Figs. 1g and 5d, specifically:

- Fig. 1g Alizarin red staining M2 image appears to overlap with Fig. 5d 100 nM miR-185-5p;
- Fig. 5d Alp staining 50 nM miR-NC and miR-185-5p images appear to overlap.

The authors provided revised figures and the underlying raw data; however, the revised figures and the associated raw data contained a further case of highly similar images between different groups.

The Editors-in-Chief therefore no longer have confidence in the presented data.

Huaiguang Chang, Hongshan Zhao and Hailan Feng disagree with this retraction. Yue Wang, Haochen Liu, Xu Nan, Singwai Wong, Saihui Peng and Yajuan Gu have not responded to any correspondence from the editor or publisher about this retraction.

